# Additive interaction between birth asphyxia and febrile seizures on autism spectrum disorder: a population-based study

**DOI:** 10.1186/s13229-024-00596-3

**Published:** 2024-04-10

**Authors:** Yi Mao, Xindi Lin, Yuhan Wu, Jiayi Lu, Jiayao Shen, Shaogen Zhong, Xingming Jin, Jun Ma

**Affiliations:** 1 Department of Developmental and Behavioral Pediatrics, Shanghai Children’s Medical Center, School of Medicine, Shanghai Jiao Tong University, Shanghai, 200127 China; 2 Department of Nephrology, Shanghai Children’s Medical Center, School of Medicine, Shanghai Jiao Tong University, Shanghai, 200127 China

**Keywords:** Autism spectrum disorder, Birth asphyxia, Children, Epidemiological study, Febrile seizures, Interaction effect

## Abstract

**Background:**

Autism Spectrum Disorder (ASD) is a pervasive neurodevelopmental disorder that can significantly impact an individual’s ability to socially integrate and adapt. It’s crucial to identify key factors associated with ASD. Recent studies link both birth asphyxia (BA) and febrile seizures (FS) separately to higher ASD prevalence. However, investigations into the interplay of BA and FS and its relationship with ASD are yet to be conducted. The present study mainly focuses on exploring the interactive effect between BA and FS in the context of ASD.

**Methods:**

Utilizing a multi-stage stratified cluster sampling, we initially recruited 84,934 Shanghai children aged 3–12 years old from June 2014 to June 2015, ultimately including 74,251 post-exclusion criteria. A logistic regression model was conducted to estimate the interaction effect after controlling for pertinent covariates. The attributable proportion (AP), the relative excess risk due to interaction (RERI), the synergy index (SI), and multiplicative-scale interaction were computed to determine the interaction effect.

**Results:**

Among a total of 74,251 children, 192 (0.26%) were diagnosed with ASD. The adjusted odds ratio for ASD in children with BA alone was 3.82 (95% confidence interval [CI] 2.42–6.02), for FS alone 3.06 (95%CI 1.48–6.31), and for comorbid BA and FS 21.18 (95%CI 9.10–49.30), versus children without BA or FS. The additive interaction between BA and FS showed statistical significance (*P* < 0.001), whereas the multiplicative interaction was statistically insignificant (*P* > 0.05).

**Limitations:**

This study can only demonstrate the relationship between the interaction of BA and FS with ASD but cannot prove causation. Animal brain experimentation is necessary to unravel its neural mechanisms. A larger sample size, ongoing monitoring, and detailed FS classification are needed for confirming BA-FS interaction in ASD.

**Conclusion:**

In this extensive cross-sectional study, both BA and FS were significantly linked to ASD. The coexistence of these factors was associated with an additive increase in ASD prevalence, surpassing the cumulative risk of each individual factor.

**Supplementary Information:**

The online version contains supplementary material available at 10.1186/s13229-024-00596-3.

## Background

Autism spectrum disorder (ASD) is a complex neurodevelopmental disorder encompassing a broad spectrum of developmental impairments, profoundly affecting an individual’s functional abilities in various domains, including social interaction, communication, and adaptive behavior [1]. A substantial body of research suggests that ASD is characterized by a widespread disruption of brain neural networks resulting from a combination of genetic and environmental factors [2–4]. Therefore, seeking important factors associated with ASD, especially those with larger effect sizes, is of significant importance for gaining a deeper understanding of the nature of ASD and its potential etiology.

Birth asphyxia (BA) is a primary neonatal pathology, often resulting in long-term cognitive deficits including learning disabilities, epilepsy, developmental delays, and intellectual disability [5]. Some cohort studies have found that children exposed to asphyxia during perinatal period were at an increased risk of developing ASD in later life [6, 7]. Febrile seizures (FS), characterized as convulsive episodes in association with fever, are distinct from seizures caused by intracranial pathologies, metabolic imbalances, or acute neurological insults [8]. Previous studies indicated that FS is related to a range of developmental and behavioral problems [9–12]. According to a nationally representative sample of twins, FS is associated with increased ASD prevalence [13]. Numerous pieces of evidence suggest that BA, as a perinatal brain injury, could compromise the early foundational structures and functions of the brain, thereby increasing its susceptibility to subsequent injuries [5, 14–16]. This vulnerability reflects a critical period wherein the developing brain is particularly sensitive to environmental insults. In comparison, FS, as postnatal factors potentially impacting brain integrity, were generally considered to have a minimal detrimental effect on a healthy brain [17, 18]. However, in brains with foundational vulnerabilities, such as those subjected to BA-induced damage during the perinatal period, the deleterious effects of FS could be significantly amplified, suggesting a heightened risk of exacerbated neurological damage in these susceptible populations. In instances where basic brain structures and functions are compromised, the subsequent occurrence of FS can be likened to a hurricane striking a house with an unstable foundation, exacerbating an already precarious situation. Therefore, even though FS typically exert a milder impact on neural integrity, their occurrence on a backdrop of BA can potentially lead to more severe neurodevelopmental injuries [19]. This interaction may result in a synergistic effect, where the combined impact is greater than the sum of the individual conditions, illustrating a ‘1 + 1 > 2’ scenario in terms of neurodevelopmental injuries. Furthermore, both BA and FS have the potential to initiate or aggravate immune dysfunction and neuroinflammation [20–24]. The concurrent presence of BA and FS might act as a ‘two-hit’ insult to the brain, culminating in extensive neurodevelopmental injuries. It is well established that extensive neurodevelopmental injuries significantly elevate the risk of ASD onset [25, 26].

Consequently, we hypothesized that BA and FS may exhibit an additive interaction, correlating with an increased prevalence of ASD. Additionally, to comprehensively understand the interplay between FS and BA and their relationship with ASD, we have also explored the presence of a potential multiplicative interaction between these factors.

## Methods

### Study design and sampling

An epidemiological survey was conducted among children aged 3–12 years old in Shanghai (population 24,197,000) from June 2014 to June 2015. Employing a stratified random cluster sampling approach, three urban districts (Yangpu, Xuhui, Jingan) from Shanghai’s seven urban districts, and four suburban districts (Pudong, Minhang, Chongming, Fengxian) from the nine suburban districts were systematically selected. This method ensured a comprehensive and representative cross-section of Shanghai’s diverse urban and suburban populations for the study. A total of 15% kindergartens and primary schools from the sampled districts were incorporated into our study, encompassing all children attending these schools as survey subjects. Recognizing that the majority of children with ASD were recruited in special education schools, all children from every special education school within the sampled districts were included to ensure comprehensive representation in our research.

**Fig. 1:**
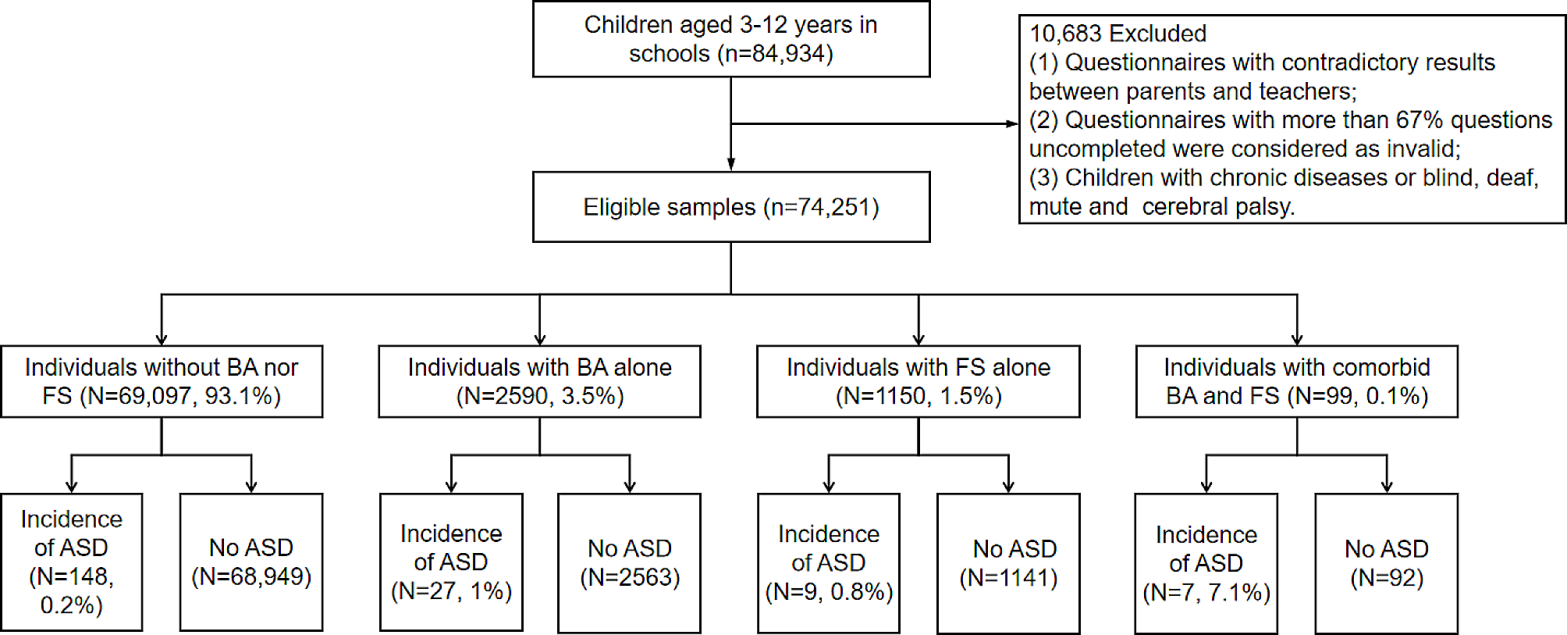
Flowchart of study population. BA: birth asphyxia; FS: febrile seizures; ASD: autism spectrum disorder

In total, 84,934 children aged 3–12 years, drawn from 96 kindergartens, 55 primary schools, and 28 special education schools, were enrolled in this study. Following the application of specific exclusion criteria to remove cases that did not meet the study’s requirements, 74,251 children were selected for the analysis (Fig. 1). The described sampling method has been thoroughly detailed in prior published papers [27, 28].

### Exposures

#### Children’s family social environment and growth questionnaire

This questionnaire, designed for investigating the growth, development, family and social environmental conditions of children, was extracted from key items of well-validated questionnaires in existing literature [29–31]. It includes a detailed survey of the child’s general health history during the fetal and perinatal periods, previous medical conditions, and a thorough assessment of family background. Key factors such as the child’s age [32], sex [33], district [34], Body Mass Index (BMI) [35], birth weight [36], gestational age [37], mode of delivery [38], feeding practices [39], parental age [40, 41], educational levels of the parents [42], parents’ personalities [43], maternal psychological status [44], complications during pregnancy [45] and the annual household income [34] are all considered. The questionnaire is comprehensive, comprising 44 items in total, each designed to capture essential data points crucial for understanding the multifaceted influences on a child’s development. For instance, the mode of delivery refers to whether the birth was vaginal or via cesarean section; parents’ personalities are categorized as introverted or extroverted, based on self-assessment and the predominant perception of others; feeding practices are defined as exclusive breastfeeding, formula feeding, or a combination of both; complications during pregnancy referred to the presence of one or more adverse factors, such as threatened abortion, medication during pregnancy, high fever (above 39℃), heart disease, intense mental stress, hyperemesis gravidarum, and anemia [30, 31]. This approach has been applied in multiple published studies, demonstrating its effectiveness and relevance in research contexts [46–48].

### Diagnostic criteria for BA and FS

BA occurs when a newborn fails to initiate and maintain spontaneous breathing at birth, potentially leading to permanent brain cell damage and posing a serious threat to the infant’s life [49]. This condition is characterized by evidence of anoxic events during birth and is diagnosed based on meeting at least two of the following criteria: an Apgar score below 5 at 5 to 10 min post-birth, the need for mechanical ventilation or resuscitation, the fetal umbilical artery acidemia, multi-system organ failure, and radiological evidence of hypoxic-ischemic encephalopathy [50]. For parents who indicated ‘yes’ to the question in the questionnaire regarding ‘oxygen deficiency or asphyxia at birth’, we conducted a comprehensive review of their child’s medical history records and imaging reports. The diagnosis was made rigorously in accordance with the established diagnostic criteria for BA.

The diagnostic criteria for FS, according to the International League Against Epilepsy (ILAE) and the International Statistical Classification of Diseases and Related Health Problems 10th Revision (ICD-10), require a child over a month old to experience a fever-related epileptic seizure, with the fever not being attributable to any central nervous system (CNS) infection. The child should have no history of neonatal seizures or prior seizures without a clear cause, and the event should not fit the profile of other specified acute symptomatic seizures [51, 52]. For parents who indicated ‘yes’ to the question in the questionnaire regarding a history of FS, we conducted a thorough review of their child’s medical history records. The diagnosis was made in accordance with the established diagnostic criteria. Due to caregivers’ apprehension of the alarming convulsions induced by FS, almost all children with FS are promptly brought to hospitals, resulting in a minimal rate of missed diagnoses.

### Outcomes

#### The social communication questionnaire (SCQ)

The SCQ, derived from Autism Diagnostic Interview-Revised (ADI-R), screens for ASD risk in children [53]; scores over 15 suggest possible ASD [54]. The Chinese version of the SCQ demonstrates a sensitivity of 0.93 and a specificity of 0.98 [55], with a false-negative rate of 1.2 per 10,000 [28]. Moreover, it demonstrates high internal consistency (a Cronbach’s alpha of 0.92), affirming its reliability in ASD screening [55].

#### ASD diagnostic procedure

Initially, Children’s Family Social Environment and Growth Questionnaire and the SCQ were distributed to parents and teachers of each child for completion. This was to screen for the potential presence of ASD. If either the parent or teacher questionnaire results in an SCQ score of 15 or higher, the child is then directed to undergo a diagnostic evaluation process for ASD.

The clinical assessment and diagnosis of ASD were conducted at the Shanghai Children’s Medical Center’s Developmental and Behavioral Pediatrics department. This process was overseen by two developmental and behavioral pediatricians with extensive clinical experience. They conducted comprehensive clinical interviews and physical examinations (with a particular focus on neurological assessments) for all children suspected of having ASD. This was followed by detailed auxiliary examinations, including electroencephalogram (EEG), cranial magnetic resonance imaging (MRI), Wechsler Intelligence Scale testing, and developmental-behavioral evaluations. The final diagnosis was in strict accordance with the Fifth Edition of the Diagnostic and Statistical Manual of Mental Disorders (DSM-5) criteria.

### Quality assurance

A comprehensive training program was implemented for researchers to thoroughly comprehend the study’s objectives, procedures, methodologies, and safety measures, ensuring uniform understanding and adherence to the research protocol. During the data collection phase, a standardized guideline was employed to instruct parents and teachers on the critical aspects of completing the questionnaires. Following collection, we scrutinized the questionnaires to pinpoint and correct any inaccuracies or gaps. This process entailed engaging with parents and teachers for essential adjustments. Moreover, all data were coded adhering to uniform standards and subjected to an anonymous screening process. Another researcher re-entered 15.0% of the questionnaire data, yielding high agreement rates, ensuring data validation. Epidata 3.1 was employed for efficient data entry and rigorous logic error verification.

### Statistical analysis

Two-sample t-tests compared ASD and normal groups. Logistic regression models were employed to calculate odds ratios (ORs) and 95% confidence interval (CI) for ASD prevalence, analyzing BA, FS, and their interaction. To adjust for demographic features, we incorporated a predetermined set of covariates, including age, sex, district, and income. Additionally, we established three models to better isolate the association between BA/FS and ASD as well as reduce potential bias:1) unadjusted (Model 1); 2) adjusted for demographic features (Model 2); 3) adjusted for demographics plus BA, FS, or ASD-related covariates (Model 3). The selection of covariates was based on factors extensively reported in the literature as having significant associations with either the independent variables (BA, FS) or the dependent variable (ASD) [32–45]. These factors may potentially alter the relationship between the independent and dependent variables within the model. Subgroup analyses were conducted to explore not only the independent associations of BA and FS with ASD, but also their interactive effects within different sex and age groups.

In line with our hypothesis, our study primarily focused on the additive interaction of BA and FS in ASD. To enhance our understanding of the BA, FS, and ASD relationship and to probe the synergistic effect where combined impact exceeds individual contributions (1 + 1 > 2), our investigation centered on assessing additive interactions. Moreover, we delved into the potential multiplicative interaction between BA and FS in ASD, aiming for a comprehensive grasp of their interconnected roles. Additive scale interaction implies that the aggregate impact of two exposures exceeds (or falls below) their individual effects summed, while multiplicative scale interaction [Eq. (4)] denotes the combined effect surpassing (or being less than) their individual impacts multiplied [56]. The relative excess risk of interaction [RERI; Eq. (1)], the attributable proportion to interaction [AP; Eq. (2)] and the synergy index [SI; Eq. (3)] are represented to calculate the results of additive interaction [57]. In Eqs. (1–4), ${\varvec{O}\varvec{R}}_{BA\&FS}$ represents the Odd Ratio (OR) for both BA and FS, ${\varvec{O}\varvec{R}}_{BA} and {\varvec{O}\varvec{R}}_{FS}$ represent the OR for BA or FS alone, respectively.

1 $${\varvec{R}\varvec{E}\varvec{R}\varvec{I}}_{Interaction}={\varvec{O}\varvec{R}}_{BA\&FS}-{\varvec{O}\varvec{R}}_{BA}-{\varvec{O}\varvec{R}}_{FS}+1$$

3 $${\varvec{A}\varvec{P}}_{Interaction}=\frac{{\varvec{O}\varvec{R}}_{BA\&FS}-{\varvec{O}\varvec{R}}_{BA}-{\varvec{O}\varvec{R}}_{FS}+1}{{\varvec{O}\varvec{R}}_{BA\&FS}}$$

3 $${\varvec{S}\varvec{I}}_{Interaction}=\frac{{\varvec{O}\varvec{R}}_{BA\&FS}-1}{[\left({\varvec{O}\varvec{R}}_{BA}-1\right)+({\varvec{O}\varvec{R}}_{FS}-1\left)\right]}$$

4 $${ \varvec{M}\varvec{u}\varvec{l}\varvec{t}}_{Interaction}=\frac{{\varvec{O}\varvec{R}}_{BA\&FS}}{{\varvec{O}\varvec{R}}_{BA}*{\varvec{O}\varvec{R}}_{FS}}$$

All analyses were performed using Statistical Program for Social Science (SPSS) software (version 26.0) and R statistics software (version 4.2.2). We considered a two-tailed *P* value of less than 0.05 as statistically significant.

## Results

### Participants characteristics and ASD prevalence

The baseline characteristics of participants were presented in Table 1. Among the initial cohort of 84,934 children, a total of 10,683 were excluded from the study for the following reasons: (1) Questionnaires exhibiting contradictory responses between parents and teachers resulted in the exclusion of 8,503 participants; (2) Questionnaires deemed invalid due to more than 67% of the questions being incomplete led to the exclusion of 2,155 participants; (3) Children diagnosed with chronic diseases or those who were blind, deaf, mute, or had cerebral palsy were excluded, accounting for 25 participants. Consequently, the study proceeded with a final sample of 74,251 participants, with a median age of 7.5 years. The cohort achieved gender balance, comprising 53.3% boys. Prevalence of BA was noted in 3.7% of cases, whereas FS affected 1.7%. Our findings of 192 participants with ASD (0.26%) were consistent with similar database research [58]. Variance Inflation Factor (VIF) was used to evaluate multicollinearity. All square roots of the VIF values were below 2, thereby excluding the possibility of high correlation among the independent variables. Figure 1 categorizes participants into four groups based on BA and FS status. The prevalence of ASD in the reference group (no BA or FS) was 0.2% (*N* = 148/69,097), 1.0% in the BA-only group (*N* = 27/2,590), 0.8% in the FS-only group (*N* = 9/1,150), and markedly higher at 7.1% in the group with both BA and FS (*N* = 7/99).

**Table 1:** The baseline characteristics of the participants

Variables	Total (*N* = 74,251)	**ASD**		Crude OR^e^ 95%CI, *P* value	Adjusted OR^f^ 95%CI, *P* value
		Present (*N* = 192)	Absent (*N* = 74059)		
Birth asphyxia, No. (%)	2689(3.7)	34(17.8)	2655(3.6)	5.72(3.94–8.30), P < 0.001	3.64(2.30–5.75), P < 0.001
Febrile seizures, No. (%)	1273(1.7)	16(8.3)	1257(1.7)	5.27(3.15–8.81), P < 0.001	3.68(2.04–6.64), P < 0.001
Age, Median (IQR), years old	7.50 (3.8)	8.50 (4.2)	7.50 (3.8)	1.01(1.00-1.02), P < 0.001	1.01(1.01–1.02), P < 0.001
Sex, boys, No. (%)	39,033 (53.3)	145 (75.5)	38,888 (52.5)	2.84(2.03–3.96), P < 0.001	2.77(1.87–4.10), P < 0.001
BMI^a^, kg/m2, mean (SE)	16.98(3.7)	17.96(4.0)	16.97(3.7)	1.05(1.03–1.09), P < 0.001	1.04(1.00-1.08), P = 0.075
District, No. (%)					
Suburb	58,312(78.6)	139(72.4)	58,173(78.6)	1[Reference]	1[Reference]
Urban	15,908(21.4)	53(27.6)	15,855(21.4)	1.40(1.02–1.92), P = 0.038	0.84(0.57–1.24), P = 0.374
Birth weight (grams), No. (%)					
2,500-4,000	60,560(84.4)	163(86.7)	60,397(84.4)	1[Reference]	
< 2,500	3492(4.9)	10(5.3)	3482(4.9)	1.06(0.56–2.02), P = 0.849	
> 4,000	7712(10.7)	15(8.0)	7697(10.8)	0.72(0.43–1.23), P = 0.722	
Gestational age, No. (%)					
37-42w	65,818(90.2)	165(87.3)	65,653(90.2)	1[Reference]	1[Reference]
< 37w	4340(5.9)	18(9.5)	4322(5.9)	1.66(1.01–2.70), P = 0.042	1.07(0.60–1.91), P = 0.816
> 42w	2786(3.8)	6(3.2)	2780(3.8)	0.86(0.38–1.94), P = 0.714	1.20(0.53–2.76), P = 0.661
Mode of delivery, No. (%)					
Vaginal	34,932(47.6)	76(40.4)	34,856(47.6)	1[Reference]	1[Reference]
Cesarean	38,417(52.4))	112(59.6)	38,305(52.4)	1.34(1.00-1.80), p = 0.049	1.08(0.76–1.52), P = 0.677
Feeding practices, No. (%)					
Breast	36,361(49.5)	69(36.1)	36,292(49.5)	1[Reference]	1[Reference]
Formula	11,817 (15.9)	46(24.1)	11,771(16.1)	2.06(1.42–2.99), P < 0.001	1.71(1.10–2.65), P = 0.016
Mixed	25,309(34.4)	76(39.8)	25,233(34.4)	1.58(1.14–2.20), P = 0.006	1.07(0.72–1.58), P = 0.750
Paternal age^b.^ No. (%)					
< 35 years	59,602(87.2)	144(82.8)	59,458(87.2)	1[Reference]	1[Reference]
≥ 35 years	8774(12.8)	30(17.2)	8744(12.8)	1.42(0.96–2.10), P = 0.083	1.13(0.71–1.79), P = 0.601
Maternal age^b^. No. (%)					
< 35 years	68,037(95.0)	178(94.2)	67,859(95.0)	1[Reference]	1[Reference]
≥ 35 years	3562(5.0)	11(5.8)	3551(5.0)	1.18(0.64–2.17), P = 0.593	
Introverted father, No. (%)	10,676(14.6)	53(28.5)	10,623(14.6)	2.33(1.69–3.20), P < 0.001	1.98(1.37–2.86), P < 0.001
Introverted mother, No. (%)	6253(8.6)	27(14.5)	6226(8.6)	1.81(1.20–2.72), P = 0.004	1.49(0.93–2.37), P = 0.094
Paternal educational level^c^, No. (%)					
Low	21,142(28.7)	49(26.2)	21,093(28.7)	1[Reference]	1[Reference]
Middle	29,879(40.5)	70(37.4)	29,809(40.5)	2.37(1.49–3.78), P < 0.001	1.57(0.80–3.08), P = 0.194
High	22,771(30.9)	68(36.4)	22,703(30.8)	3.64(2.30–5.76), P < 0.001	2.97(1.41–6.27), P = 0.004
Maternal educational level^c^, No. (%)					
Low	24,079(32.6)	58(31.2)	24,021(32.6)	1[Reference]	1[Reference]
Middle	29,763(40.3)	73(39.2)	29,690(40.3)	2.66(1.74–4.07), P < 0.001	2.36(1.22–4.57), P = 0.011
High	19,967(27.1)	55(29.6)	19,912(27.0)	3.07(1.98–4.75), P < 0.001	2.14(1.01–4.55), P = 0.048
Maternal psychological status					
Normal	66,530(91.9)	160(89.9)	66,370(91.9)	1[Reference]	1[Reference]
Depressed	2411(3.3)	13(7.3)	2398(3.3)	3.44(2.11–5.63), P < 0.001	2.62(1.51–4.56), P = 0.001
Nervous	3449(4.8)	5(2.8)	3444(4.8)	3.07(1.98–4.78), P < 0.001	2.23(1.33–3.76), P = 0.003
Complications during pregnancy	16,239(21.9)	75(39.1)	16,164(21.8)	2.30(1.72–3.07), P < 0.001	1.82(1.28–2.59), P = 0.001
Annual Household Income^d^, No. (%)					
Low	2369(3.3)	9(4.8)	2360(3.3)	1.47(0.74–2.93), P = 0.268	
Medium-low	7188(9.9)	23(12.2)	7165(9.9)	1.24(0.78–1.96), P = 0.357	
Medium	10,583(14.6)	23(12.2)	10,560(14.6)	0.84(0.53–1.33), P = 0.460	
Medium-high	17,057(23.5)	42(22.3)	17,015(23.5)	0.95(0.66–1.38), P = 0.799	
High	35,246(48.7)	91(48.4)	35,155(48.7)	1[Reference]	

^a^ BMI: Body Mass Index, calculated as weight (kg) divided by height squared (m^2^); ^b^. Parental age denotes the chronological age of both the mother and father at the time of the mother’s confirmed pregnancy diagnosis; ^c^ Parental Educational Level was categorized as follows: Low (less than a high school diploma), Middle (high school graduate or equivalent), High (bachelor’s degree or above); ^d^ Annual Household Income was classified into the following categories: Low (< ¥10,000), Medium-Low (¥10,000–29,999), Medium (¥30,000–49,999), Medium-High (¥50,000–99,999), and High (>¥100,000); ^e^ In the univariate regression analysis, variables with a *P*-value < 0.1, including BA, FS, age, sex, BMI, district, gestational age, mode of delivery, feeding practices, paternal age, introverted father, introverted mother, paternal educational level, maternal educational level, maternal psychological status, and complications during pregnancy were included in the multivariate model; ^f^ In the multivariate regression analysis, the presence of BA, FS, age, sex, feeding practices, introverted father, paternal educational level, maternal educational level, maternal psychological status, and complications during pregnancy were significantly associated with ASD (*P* < 0.05)

### The association between BA/FS and ASD occurrence

A significant association of BA and FS with increased ASD prevalence was found in Table 2. The analysis indicated a significant link between BA/FS and an increased prevalence of ASD, as detailed in Table 2. In the fully adjusted model (Model 3), participants with BA were associated with a 290% increased prevalence of ASD (OR 3.90, 95%CI 2.60–5.86) compared to participants without BA. Similarly, participants with FS showed a 252% increased prevalence (OR 3.52, 95%CI 2.03–6.10) relative to participants without FS. Deeper subgroup analysis factoring in age and sex revealed that the association between BA and ASD was particularly pronounced in participants aged 7–10 years (OR 4.71, 95%CI 2.57–8.65), and even more so in girls (OR 7.51, 95%CI 3.63–15.50), when in contrast to the correlations observed in age groups 3–7 and 10–12 years, as well as in boys. It was noteworthy that FS, a significant association with ASD was also observed in the 7–10 year age group (OR 6.02, 95%CI 2.78–13.05) and in girls (OR 3.62, 95%CI 1.08–12.12). Pertaining to the stratification by sex and age, detailed sample sizes and the corresponding effect sizes for BA, FS, and ASD across the three models are comprehensively presented in Additional file 1: Table S1.

**Table 2:** Logistic regression: associations between BA/FS and ASD

	BA-ASD Relationship Analysis			FS-ASD Relationship Analysis	
	No BA	BA		No FS	FS
Total population	70,247(96.3%)	2689(3.7%)		72,978(98.3%)	1273(1.7%)
ASD events	157(82.2%)	34(17.8%)		176(91.7%)	16(8.3%)
Model 1^a^ (OR, 95%CI, P)	1[Reference]	5.72(3.94–8.30), *P* < 0.001		1[Reference]	5.27(3.15–8.81), *P* < 0.001
Model 2^b^ (OR, 95%CI, P)	1[Reference]	5.35(3.66–7.82), *P* < 0.001		1[Reference]	4.81(2.83–8.20), *P* < 0.001
Model 3^c^ (OR, 95%CI, P)	1[Reference]	3.90(2.60–5.86), *P* < 0.001		1[Reference]	3.52(2.03–6.10), *P* < 0.001
**Age (years)** ^**d**^					
3–7 (OR, 95%CI, P)	1[Reference]	3.71(1.78–7.72), *P* < 0.001		1[Reference]	0.55(0.07-4.00), *P* = 0.662
7–10 (OR, 95%CI, P)	1[Reference]	4.71(2.57–8.65), *P* < 0.001		1[Reference]	6.02(2.78–13.05), *P* < 0.001
10–12 (OR, 95%CI, P)	1[Reference]	3.11(1.34–7.20), *P* = 0.002		1[Reference]	5.69(2.24–4.45), *P* < 0.001
**Sex** ^**e**^					
Boys (OR, 95%CI, P)	1[Reference]	3.03(1.85–4.96), *P* < 0.001		1[Reference]	3.54(1.91–6.56), *P* < 0.001
Girls (OR, 95%CI, P)	1[Reference]	7.51(3.63–15.50), *P* < 0.001		1[Reference]	3.62(1.08–12.12), *P* = 0.033

^a^ Unadjusted; ^b^ Adjusted for demographic features such as age, sex, district, and income; ^c^ Adjusted for demographic factors and covariates related to BA, FS, or ASD, which include age, sex, district, income, feeding practices, introverted father, paternal educational level, maternal educational level, maternal psychological status, and complications during pregnancy; ^d^ Age-specific subgroup analysis: examines the link between BA/FS and ASD across different age groups, with all results adjusted using Model 3; ^e^ Sex-specific subgroup analysis: explores the association between BA/FS and ASD across sex, with all results adjusted using Model 3

### Interaction effect of BA and FS in relation to ASD

In Table 3, our analysis revealed that, relative to the reference group without BA or FS, the BA-only group demonstrated a significantly increased prevalence of ASD with OR of 3.82 (95%CI 2.42–6.02). Similarly, the FS-only group exhibited an OR of 3.06 (95%CI 1.48–6.31), indicating a heightened ASD prevalance. Most notably, the group with comorbid BA and FS showed a markedly elevated ASD prevalence with an OR of 21.18 (95%CI 9.10–49.30). The additive interaction indices for BA and FS in Model 3 were statistically significant (AP 0.72, 95%CI 0.24–0.86; RERI 15.30, 95%CI 2.95–43.27; SI 4.14, 95%CI 1.49–11.46). The AP suggests that a portion of 72% ASD risk in children with both BA and FS can be attributed to the additive interaction of these two factors. A positive RERI indicates a significant excess risk of ASD due to the combined effect of BA and FS over their individual effects and the value of SI greater than 1 demonstrates a synergistic effect between BA and FS, where their combined effect on ASD risk is greater than the sum of their individual effect. The additive interaction effect showed positively statistical significance in age 7–10 years (AP 0.85, 95%CI 0.36–0.93; RERI 46.83, 95%CI 8.04-173.02; SI 7.37, 95%CI 1.79–30.36) and girls (AP 0.90, 95%CI 0.36–0.95; RERI 74.72, 95%CI 8.76-394.17; SI 11.87, 95%CI 1.95–72.24). Given that all multiplicative interaction intervals included 1, aligning with the null hypothesis, there was insufficient evidence to demonstrate a significant multiplicative interaction between BA-FS and ASD in this study.

**Table 3:** Interaction effect of BA and FS in relation to ASD

	Odds ratios of exposure (95%CI)				Additive interaction (95%CI)^e^			Multiplicative interaction (95%CI)^f^
	No BA/FS	BA	FS	BA and FS	AP	RERI	SI	
Model 1^a^	1[Reference]	4.91(3.25–7.41) ***	3.68(1.87–7.22) ***	35.45(16.17–77.73) ***	0.79(0.46–0.89)	27.86(8.43-70.00)	5.23(2.09–13.09)	1.97(0.66–5.85)
Model 2^b^	1[Reference]	4.56(2.99–6.95) ***	3.22(1.57–6.58) **	29.45(13.26–65.42) ***	0.77(0.40–0.88)	22.67(6.26–58.52)	4.93(1.91–12.73)	2.01(0.65–6.21)
Model 3^c^	1[Reference]	3.82(2.42–6.02) ***	3.06(1.48–6.31) **	21.18(9.10–49.30) ***	0.72(0.24–0.86)	15.30(2.95–43.27)	4.14(1.49–11.46)	1.81(0.56–5.82)
**Age (years)**								
3–7	1[Reference]	4.14(1.98–8.65) ***	0.86(0.12–6.28) *	N/A^d^	N/A	N/A	N/A	N/A
7–10	1[Reference]	4.78(2.40–9.52) ***	5.16(1.81–14.66) **	67.47(20.61-220.84) ***	0.85(0.36–0.93)	46.83(8.04-173.02)	7.37(1.79–30.36)	2.74(0.51–14.63)
10–12	1[Reference]	2.17(0.73–6.50) *	4.59(1.32–15.94) **	28.22(6.68-119.23) ***	0.78(-0.28-0.90)	22.52(-1.26-113.61)	5.33(0.87–32.58)	2.83(0.34–23.43)
**Sex**								
Boys	1[Reference]	2.86(1.63–5.03) ***	3.21(1.47-7.00) **	15.65(5.80-42.21) ***	0.68(-0.08-0.83)	10.58(0.13–37.01)	3.60(1.04–12.51)	1.71(0.45–6.53)
Girls	1[Reference]	7.15(3.20-15.99) ***	1.72(0.23–13.17) *	82.60(16.95-402.51) ***	0.90(0.36–0.95)	74.72(8.76-394.17)	11.87(1.95–72.24)	6.71(0.47–96.51)

* *P* > 0.05, ** *P* < 0.05, *** *P* < 0.001; ^a^ Unadjusted; ^b^ Adjusted for demographic features such as age, sex, district, and income; ^c^ Further adjusted for demographic and BA, FS, ASD-related covariates, including age, sex, district, income, feeding practices, introverted father, paternal education level, maternal education level, maternal psychological status, and complications during pregnancy; ^d^ N/A denotes an insufficient sample size for result calculation; ^e^ The null hypothesis for each interaction: AP = 0, RERI = 0, SI = 1; ^f^ The null hypothesis for each interaction: the multiplicative interaction = 1 (All *P* > 0.05 in this multiplicative interaction analysis)

## Discussion

This study marked the initial foray into a population-based epidemiological analysis focusing on the interplay between BA and FS in the context of ASD. Post-adjustment for covariates, we discerned a significant additive interaction between BA and FS related to ASD prevalence (AP 0.72, 95%CI 0.24–0.86; RERI 15.30, 95%CI 2.95–43.27; SI 4.14, 95%CI 1.49–11.46). In Model 3, which controlled for as many covariates as possible, the OR for the interaction between BA and FS in association with ASD was 21.18 (95%CI 9.10–49.30). This indicated that in the population of children with both BA and FS, the prevalence of ASD was 21.18 times higher compared to the population without BA and FS. This was consistently validated through analysis using three diverse and robust statistical models (Table 3).

Nevertheless, it is crucial to emphasize that our study primarily identified the correlation between the interplay of BA and FS in relation to ASD, without asserting a causal relationship. Given the intricacy of these mechanisms, there were three possible explanations that merit consideration:

1) The additive interaction between BA and FS might result in a more pronounced impairment of the developing neural networks in children, ultimately leading to the onset of ASD.

BA typically inflicts extensive and severe damage on the brain, with the cerebral cortex and hippocampus being particularly vulnerable [59–61]. This damage was evidenced by reduced arborization in the cerebellum and cortex, a marked decrease in Purkinje cells, and a thinning of both the orienting and pyramidal layers in the dorsal hippocampus [59–61]. Such neuroanatomical changes were consistent with those identified in ASD, as supported by findings from both animal models and human neuroimaging studies [62]. Additionally, early life trauma induced by BA could lead to the recruitment of astrocytes and microglia at the injury site [63]. This response triggered alterations in neural circuit excitability, effectively lowering the brain’s threshold for excitation. Consequently, this heightened sensitivity could precipitate seizures, particularly when the brain is subjected to further stressors, such as ‘fever’ [64]. Moreover, research indicated that FS also exerted varying degrees of influence on the development of the CNS [65, 66]. In a recent study, it was observed that FS could lead to notable changes in the neural connectivity, specifically between the bilateral temporal lobes and the thalamus in children [22]. This included an increased dissociation and diminished integration of select subcortical structures, as well as alterations in the right frontal lobes. Concurrently, prolonged exposure to certain FS might induce enduring changes in dendritic complexity, alongside abnormal neuronal gene expression and the growth of excitatory synapses [67].

Due to the vulnerability induced by early brain damage from BA, the brain became more susceptible to even relatively mild risk factors, such as FS. This susceptibility could lead to further significant damage to the brain’s neural networks and adversely impact the development of CNS. However, in cases where the fundamental neuroanatomical structures and functions remain intact, certain milder risk factors, such as FS, might struggle to inflict significant damage to the brain [68]. It is predominantly in brains where the foundational structures and functionalities were compromised and exhibited vulnerability, that the deleterious effects of FS might manifest [69]. This could lead to a synergistic interaction, resulting in more severe consequences for the development of the nervous system. Moreover, a substantial body of research has established that abnormalities in the development of the CNS were a significant contributing factor in the etiology of ASD [70–72]. Therefore, it is highly plausible that BA and FS interact synergistically, substantially elevating the risk of ASD development.

2) The progression of ASD might contribute to an increased prevalence of both BA and FS, thereby intensifying the interactive dynamic between these two factors.

Research conducted by Gayle C and colleagues has identified that children with ASD detected in prenatal screenings exhibited distinctive biochemical markers, notably lower levels of unconjugated estriol (uE3) and higher concentrations of maternal serum alpha-fetoprotein (MSAFP) [73]. The uE3 levels during gestation were indicative of both fetal and placental health. Diminished uE3 has been correlated with various adverse pregnancy outcomes, including preeclampsia, preterm birth, infants small for gestational age, and compromised placental function [74]. In a similar vein, elevated MSAFP levels served as a significant prognosticator for adverse maternal/fetal health outcomes, potentially leading to the emergence of BA [75]. Furthermore, ASD encompassed variations in neurodevelopment and brain connectivity, which might render the brain more vulnerable to perturbations induced by fever, potentially precipitating FS [76–80]. Additionally, in individuals with ASD, there was often a dysregulated immune response to infections, commonly associated with fever, which might elevate the risk of FS. This altered immune reactivity in ASD could exacerbate the neurological impact of common infections, thereby increasing the likelihood of FS [81]. Therefore, ASD itself might contribute to an increased prevalence of BA and FS. This heightened occurrence served as a foundation for the amplified synergistic interaction between BA and FS.

3) Specific genetic anomalies might concurrently heighten the risk of BA, FS, and ASD; consequently, these genetic factors collectively might contribute to an elevated prevalence of BA, FS, and ASD, thus reinforcing their interplay.

Previous studies have documented that deletions and mutations in genes such as DYRK1A, SCN9A, SCN1A, and CACNA1A could lead to both ASD and FS [82–85]. Additionally, as genetic research progresses, it’s likely that more genetic defects contributing to the increased risk of developing ASD, FS, and BA would be identified. Under the influence of these genetic factors, an increased prevalence of these conditions might intensify their interplay. This represents a significant causal mechanism pathway that should not be overlooked.

This study found that the interaction between BA and FS in association with ASD demonstrated an additive interaction, with no evidence of a multiplicative interaction. Within the ‘interaction continuum’ theory, interactions were categorized into 11 levels of varying strengths [86]. In this continuum, the strongest positive interaction is positive-multiplicative positive-additive. The interaction between BA and FS in relation to ASD is classified as no-multiplicative positive-additive, ranking it as the second strongest. Therefore, from a biological and clinical medical perspective, this significant interaction warrants further in-depth investigation and should not be overlooked. Given the strong interaction between BA and FS associated with ASD, this study carries significant clinical implications. It suggests that for children clinically suspected of ASD, a detailed history regarding the presence of BA and FS should be meticulously assessed. Particularly, children exhibiting both BA and FS warrant intensified screening and monitoring for ASD.

In our study, the prevalence of ASD in the assessed population was approximately 0.26%, which was below the global estimate of 0.76% as reported by the World Health Organization (WHO) [87]. A systematic review reported an ASD prevalence of 0.34% (95%CI 0.08%-1.4%) [88] in Southeast Asian populations. A meta-analysis reported ASD prevalence in China, including Hong Kong and Taiwan regions, at 0.27% (95%CI 0.19%-0.35%) [89]. Furthermore, the most extensive cross-sectional epidemiological study on ASD to date, encompassing children aged 6–12 across eight representative cities in China, reported a prevalence of 0.29% (95%CI 0.26%-0.32%) [90]. Consequently, the ASD prevalence observed in our study aligned with the majority of research conducted in China but was lower than the rates reported in some other countries [91]. Potential reasons for this discrepancy could include significant variations in ASD prevalence across different ethnic populations. Additionally, our study adhered strictly to the DSM-5 diagnostic criteria for ASD, which tended to identify fewer cases compared to the DSM-4 criteria [92].

Furthermore, in a multivariable logistic regression model, we identified associations with ASD that align with findings reported in prior studies. These associations included age [93], sex [94], feeding practices [39], introverted father [95], paternal educational level [96], maternal educational level [96], maternal psychological status [97], and complications during pregnancy [7]. In three statistical models, both crude and adjusted ORs showed statistical significance, affirming these variables’ association with ASD, independent of other factors.

The study’s robust methodology, utilizing a large and representative sample from one of the world’s largest cities, Shanghai, is a significant strength. Including children from all special education schools within the sampled districts substantially minimized the risk of undetected ASD cases, enhancing the study’s validity and reliability. Second, the age range of the sample, from 3 to 12 years, was comprehensive, covering the typical age range for ASD diagnosis and the onset of pronounced symptoms. Third, diagnostic protocols conformed to DSM-5 and best practices, integrating parent-teacher feedback with evidence-based tools. Children with suspected ASD were referred to two seasoned developmental and behavioral pediatricians for in-depth clinical evaluations, including history, physical exams, MRI, EEG, and behavioral assessments, ensuring accurate diagnoses. Fourth, our study adjusted for numerous covariates, enabling a detailed analysis of the additive interaction between BA and FS and its association with ASD. This interaction showed a significant effect size and was consistently evident across three stringent statistical models. Fifth, given our substantial sample size, we also explored this BA-FS interaction in relation to ASD across different age and sex strata, enhancing the robustness of our findings.

### Limitations

Several key limitations warrant attention. First, based on the evidence presented in this paper, we can only assert a notable association between the interaction of BA and FS, and their correlation with ASD. However, this did not establish causality between these factors, nor did it elucidate the underlying mechanisms. Second, given the cross-sectional nature of the research, we were unable to trace longitudinal changes in participants, thereby lacking precise information on the timing of events. This limitation hampers understanding of the long-term cumulative effects of BA and FS on the risk trajectory for ASD. Third, SCQ screening phase might have overlooked some ASD cases, but considering the SCQ’s role as a standardized screening instrument, the rate of missed diagnoses is remarkably low, at only 1.2 per 100,000 [28]. Fourth, while we noted a more pronounced effect of the BA and FS interaction linked to ASD in children aged 7–10 and girls, the broad and overlapping 95%CI suggested uncertainty in these age and sex-specific differences. In addition, our study’s low ASD prevalence meant that, despite a large sample, only 7 children with BA and FS were diagnosed with ASD, hence the OR values were susceptible to fluctuations in case numbers. Thus, our findings necessitate replication through multicenter, multicity, and nationwide studies with larger samples for more reliable conclusions. Fifth, our epidemiological study did not encompass genetic testing for each participant, their parents, or information on the BA, FS, and ASD conditions among first- and second-degree relatives. This omission presents a significant challenge in fully accounting for genetic influences. Future research is needed to unravel the potential genetic contributions to these correlations. Sixth, while our study identified the significant additive interaction between BA and FS in ASD, it did not explore their interplay with broader neurodevelopmental or physical health domains. This area awaits exploration in future research.

## Conclusion

This study highlighted a robust synergistic interplay between BA and FS as they pertain to ASD. These insights are pivotal for the progression of ASD research and clinical practice, guiding us to: 1) offer clues for deeper investigation into the causes of ASD; 2) assess the detailed history of BA and FS in children clinically suspected of ASD; and 3) emphasize the critical need for vigilant ASD or developmental disorders screening and surveillance in children with BA and FS histories.

### Electronic supplementary material

Below is the link to the electronic supplementary material.

**Supplementary Material 1: Table S1.** Logistic regression: associations between BA/FS and ASD, including age and sex stratification in all three statistical models

## Data Availability

The data that support the findings of this study are available on request from the corresponding author, JM. The data are not publicly available due to privacy restrictions.

## Abbreviations

ADI: Autism Diagnostic Interview
AP: Attributable Proportion
ASD: Autism Spectrum Disorder
BA: Birth Asphyxia
BMI: Body Mass Index
CI: Confidence Interval
CNS: Central Nervous System
DSM-5: The Fifth Edition of the Diagnostic and Statistical Manual of Mental Disorders
EEG: Electroencephalogram
FS: Febrile Seizures
ICD-10: International Statistical Classification of Diseases and Related Health Problems 10th Revision
ILAE: The International League Against Epilepsy
MRI: Magnetic Resonance Imaging
MSAFP: Maternal Serum Alpha-Fetoprotein
ORs: Odd Ratios
RERI: Relative Excess Risk of Interaction
SCQ: Social Communication Questionnaire
SI: Synergy Index
SPSS: Statistical Program for Social Science
UE3: Unconjugated Estriol
VIF: Variance Inflation Factor
WHO: World Health Organization
