# Supplementary material for: Additive interaction between birth asphyxia and febrile seizures on autism spectrum disorder: a population-based study
Source: Mol Autism. 2024 Apr 10;15:17. doi: 10.1186/s13229-024-00596-3 (PMC11007945; doi:10.1186/s13229-024-00596-3)
Supplement: Supplementary file 1 — Supplementary Material 1: Table S1. Logistic regression: associations between BA/FS and ASD, including age and sex stratification in all three statistical models [file 13229_2024_596_MOESM1_ESM.pdf]

Additional file 1

Table S1 Logistic regression: associations between BA/FS and ASD, including age and sex stratification in all three statistical models

	**BA-ASD Relationship Analysis**		**FS-ASD Relationship Analysis**	
	**No BA**	**BA**	**No FS**	**FS**
Total population [n (%)]	70,247(96.3%)	2689(3.7%)	72,978(98.3%)	1273(1.7%)
ASD events [n (%)]	157(82.2%)	34(17.8%)	176(91.7%)	16(8.3%)
**Age (years)[d]**				
3-7 [n (%)]	30,980(96.6%)	1091(3.4%)	31,865(98.0%)	652(2.0%)
ASD events [n (%)]	52(85.2%)	9(14.8%)	60(98.4%)	1(1.6%)
Model 1[a] (OR, 95%CI, P)	1[Reference]	4.95(2.43-10.07), P<0.001	1[Reference]	0.81(0.11-5.88), P=0.839
Model 2[b] (OR, 95%CI, P)	1[Reference]	4.59(2.25-9.36), P<0.001	1[Reference]	0.76(0.11-5.49), P=0.778
Model 3[c] (OR, 95%CI, P)	1[Reference]	3.71(1.78-7.72), P<0.001	1[Reference]	0.55(0.07-4.00), P=0.662
7-10 [n (%)]	25,828(96.2%)	1026(3.8%)	26,938(98.5%)	420(1.5%)
ASD events [n (%)]	59(77.6%)	17(22.4%)	69(89.6%)	8(10.4%)
Model 1 (OR, 95%CI, P)	1[Reference]	7.36(4.28-12.67), P<0.001	1[Reference]	7.56(3.61-15.82), P<0.001
Model 2 (OR, 95%CI, P)	1[Reference]	6.62(3.79-11.58), P<0.001	1[Reference]	7.29(3.47-15.32), P<0.001
Model 3 (OR, 95%CI, P)	1[Reference]	4.71(2.57-8.65), P<0.001	1[Reference]	6.02(2.78-13.05), P<0.001
10-12 [n (%)]	12,881(95.9%)	545(4.1%)	13,477(98.6%)	194(1.4%)
ASD events [n (%)]	45(84.9%)	8(15.1%)	46(86.8%)	7(13.2%)
Model 1 (OR, 95%CI, P)	1[Reference]	4.25(1.99-9.06), P<0.001	1[Reference]	10.93(4.87-24.52), P<0.001
Model 2 (OR, 95%CI, P)	1[Reference]	4.43(2.06-9.52), P<0.001	1[Reference]	8.65(3.60-20.76), P<0.001
Model 3 (OR, 95%CI, P)	1[Reference]	3.11(1.34-7.20), P=0.002	1[Reference]	5.69(2.24-4.45), P<0.001
**Sex[e]**				
Boys [n (%)]	36,821(95.9%)	1589(4.1%)	38,260(98.0%)	773(2.0%)
ASD events [n (%)]	123(84.8%)	22(15.2%)	133(91.7%)	12(8.3%)
Model 1 (OR, 95%CI, P)	1[Reference]	4.19(2.65-6.61), P<0.001	1[Reference]	4.52(2.49-8.20), P<0.001
Model 2 (OR, 95%CI, P)	1[Reference]	4.03(2.53-6.44), P<0.001	1[Reference]	4.65(2.56-8.45), P<0.001
Model 3 (OR, 95%CI, P)	1[Reference]	3.03(1.85-4.96), P<0.001	1[Reference]	3.54(1.91-6.56), P<0.001
Girls [n (%)]	32,675(96.9%)	1060(3.1%)	33,777(98.6%)	486(1.4%)
ASD events [n (%)]	32(72.7%)	12(27.3%)	42(93.3%)	3(6.7%)
Model 1 (OR, 95%CI, P)	1[Reference]	11.68(6.00-22.74), P<0.001	1[Reference]	4.99(1.54-16.15), P<0.001
Model 2 (OR, 95%CI, P)	1[Reference]	11.71(5.99-22.90), P<0.001	1[Reference]	5.43(1.67-17.64), P=0.005
Model 3 (OR, 95%CI, P)	1[Reference]	7.51(3.63-15.50), P<0.001	1[Reference]	3.62(1.08-12.12), P=0.033

[a] Unadjusted; [b] Adjusted for demographic features such as age, sex, district, and income; [c] Adjusted for demographic factors and covariates related to BA, FS, or ASD, which include age, sex, district, income, feeding practices, introverted father, paternal educational level, maternal educational level, maternal psychological status, and complications during pregnancy; [d] Age-specific subgroup analysis: examines the link between BA/FS and ASD across different age groups, with all results adjusted using Model 3; [e] Sex-specific subgroup analysis: explores the association between BA/FS and ASD across sex, with all results adjusted using Model 3.
